# Is Capsaicin Effective in Relieving the Symptoms of Burning Mouth Syndrome? A Systematic Review

**DOI:** 10.1111/joor.70275

**Published:** 2026-07-24

**Authors:** Ana Paula de Andrade Silva, Ian Wesley Rocha dos Santos, Zuleni Alexandre da Silva, Deborah Ribeiro Frazão, José Mário Matos‐Sousa, Wallacy Watson Pereira Melo, Rafael Rodrigues Lima, Renata Duarte de Souza‐Rodrigues

**Affiliations:** ^1^ Laboratory of Functional and Structural Biology, Institute of Biological Sciences Federal University of Pará Belém Brazil

**Keywords:** burning mouth syndrome, capsaicin, chronic pain, pain management, systematic review, therapy

## Abstract

**Background:**

Burning Mouth Syndrome (BMS) is a type of chronic orofacial pain characterised by a burning sensation inside the mouth without an apparent cause. Capsaicin has been used to manage the symptoms of this syndrome, but there is still no consensus on its effectiveness.

**Objective:**

To assess whether capsaicin, when used topically or systemically, is able to alleviate the symptoms of BMS.

**Methods:**

An extensive search was carried out in seven databases and grey literature up to November 2025. There were no restrictions regarding the date or language of publication.

**Results:**

The search resulted in 782 articles, of which 4 were included. The methodological quality of the studies was assessed using the Risk of Bias in Systematic Reviews (ROBIS) tool and the certainty of evidence was performed according to Grading of Recommendations, Assessment, Development and Evaluation (GRADE). Regarding capsaicin administration, 3 studies used topical application and 1 used systemic application. Concentrations ranged from 0.000354% to 0.25%, three times a day, for 1–8 weeks. Regardless of the administration method, a reduction in patient‐reported pain scores was observed. Risk of bias assessment showed that three randomised studies presented a moderate risk, while the only non‐randomised clinical trial presented a critical risk. The overall certainty of the evidence was low or very low for the outcomes assessed.

**Conclusion:**

Evidence suggests that capsaicin is effective in relieving BMS symptoms. However, this result should be interpreted with caution due to the low quality of the evidence obtained to support its use.

## Graphical abstract

The capsaicin, administered topically or systemically, reduces pain symptoms in patients with Burning Mouth Syndrome (BMS). However, despite consistent clinical improvement, the current evidence is limited and of low certainty, highlighting the need for well‐designed studies to confirm its effectiveness.

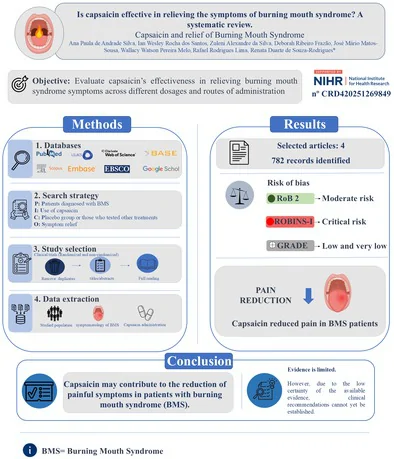

## 1 Introduction

Burning mouth syndrome (BMS) is described in the International Classification of Orofacial Pain (ICOP) as “An intraoral burning or dysesthesia sensation, recurring daily for more than 2 hours per day for more than 3 months, without evident causative lesions on clinical examination and investigation” [1]. The main symptom of this is a sensation of pain or discomfort, with bilateral burning, mainly affecting the tip and lateral edges of the tongue [2]. It may be associated with other symptoms, such as subjective xerostomia, dysgeusia, halitosis, bitter/metallic taste and even subjective changes in the colour or morphology of the tongue [3].

The prevalence of this condition varies between 0.1% and 3.9% of the general population, with most cases occurring in women over 50 years of age, usually after menopause [2]. The aetiology of BMS is not yet fully understood, but it is believed to be associated with neuropathic, endocrine and psychosocial factors, such as anxiety, depression and stress [4]. Various classifications have been proposed for BMS. Traditionally, it was classified as primary, when there was no associated disease; and secondary, which is associated with a pre‐existing condition [5, 6]. However, with the publication of the ICOP in 2020 [1], the term BMS came to be used exclusively for idiopathic cases, whereas conditions previously classified as secondary BMS are now described as oral mucosal pain attributed to a causative condition [1].

Due to the diversity of symptoms presented by patients, diagnosis becomes complex, which can lead to late initiation of treatment [7]. Therefore, the diagnostic process should begin with the identification of possible local or systemic causal factors, since this investigation is of fundamental importance for choosing the most appropriate treatment, whether topical, systemic and/or behavioural strategies [8]. In cases of pain in the oral mucosa, treatment of the associated clinical condition may result in improvement or disappearance of symptoms [5, 6, 8]. Several drugs have been used to treat or reduce the symptoms of BMS, including anticonvulsants, antidepressants and herbal components [9], however, there are still no widely accepted scientific guidelines for its treatment [5, 10].

Capsaicin (trans‐8‐methyl‐N‐vanillyl‐6‐nonenamide), a natural alkaloid derived from chilli peppers, is among the compounds used in the treatment of BMS [9]. It has been employed due to its ability to activate the transient receptor potential vanilloid 1 (TRPV1) receptor, modulating the nociceptive transmission of pain from the peripheral to the central nervous system [11, 12]. This effect occurs through the blocking of axial impulses, limiting neuropeptides and reducing the action potential of the cell membrane [13]. As a result, it is postulated that capsaicin may contribute to relieving the sensation of oral burning and pain due to desensitisation of nociceptive fibres [7, 11, 12].

Although previous studies have shown that capsaicin can alleviate BMS symptoms [14, 15, 16], some questions remain unanswered, such as whether there is a specific protocol that provides better and longer‐lasting results in relation to pain, discomfort, sensory and salivary changes. A previous systematic review investigated the effects of topical capsaicin application on BMS [14], evaluating only clinical studies with or without a control agent that used mouthwashes or gels for treatment, and concluded that capsaicin, when used in the form of a mouthwash, has potential for application in the treatment of symptoms related to BMS. Furthermore, other systematic reviews [9, 17, 18] have conducted broader analyses of BMS interventions and the pharmacological treatment of orofacial pain, identifying capsaicin as a promising alternative for pain management. However, those studies evaluated capsaicin merely as one of several therapeutic modalities without specifically exploring aspects such as the route of administration, dosage, treatment duration and therapeutic protocols. Moreover, they highlighted that available evidence remains limited, heterogeneous and of low methodological quality, precluding more robust clinical recommendations. In contrast, in order to allow a more comprehensive analysis, the present systematic review included randomised and non‐randomised clinical trials with control groups that evaluated the topical and systemic routes of administration of capsaicin. Thus, the aim of our study was to systematically evaluate whether capsaicin administration is effective in reducing the symptoms of burning mouth syndrome (BMS), whether related to pain, sensory changes, or salivary flow.

## 2 Methods

### 2.1 Registration Protocol

This systematic review followed the PRISMA (Preferred Reporting Items for Systematic Review and Meta‐Analysis) guidelines [19] and was registered in PROSPERO (protocol number CRD420251269849). The PRISMA checklist followed is contained in the Supporting Information.

### 2.2 Search Strategy

On November 6, 2025, an extensive search was conducted in the following databases: PubMed Medline, Scopus, Web of Science, Lilacs (BVS), EBSCO, Embase. To access the grey literature, Google Scholar and Base were used. Additionally, the search was conducted in two clinical trial registries: Brazilian Registry of Clinical Trials (ReBEC) and ClinicalTrials. The search strategy was developed based on MeSH terms, combining them with Boolean operators (AND or OR), and peer‐reviewed according to the criteria of the Peer Review of Electronic Search Strategies (PRESS) checklist. The search strategy was specifically tailored for each platform as described in Table S1. Also, a search alert was activated in each database to notify the inclusion of new articles that met the search criteria.

All articles found were imported into reference management software (EndNote, version X9, Thomson Reuters, Philadelphia, USA). Duplicates were deleted both manually and automatically.

### 2.3 Eligibility Criteria

The inclusion criteria adopted were randomised and non‐randomised clinical trials in which patients were diagnosed with BMS, characterised by no causative lesions evident on clinical examination and investigation [1]. Studies without a control group were also excluded, as the presence of this group is strongly recommended to allow for adequate comparison of the efficacy of capsaicin with other interventions or with placebo. No restrictions were applied regarding the publication date or language of the article. Studies conducted on animals, in vitro studies, case reports, case series, narrative reviews, descriptive studies, opinion articles, technical articles, editorials, letters to the editor and book chapters were excluded.

In order to ensure compliance with eligibility criteria and appropriate study selection, this systematic review followed the PICO acronym, which “P” refers to patients diagnosed with BMS; “I” is the use of capsaicin; “C” includes groups that did not use capsaicin, placebo or that used other treatment methods; and “O” indicates symptom relief.

### 2.4 Study Selection

The study selection process was carried out by two authors (A.P.A.S. and Z.A.S.). The results of both examiners were compared and in cases of disagreement, a third examiner was consulted (R.D.S.‐R.). After removing duplicates, the papers were analysed by reading titles and abstracts, considering the eligibility criteria. Subsequently, the resulting papers were read in full, and only those that met the objective of our study were selected. Those that were unavailable or could not be found were requested by email from the corresponding authors, and those found in languages in which the authors are not proficient were translated using online tools. In addition, the references of all the included articles were manually searched to retrieve possible studies that might have been lost in the search strategy. The articles selected and included in this study were tabulated and further explored in Table 1.

**TABLE 1:** Characteristics of the selected studies.

Author (Year) Country	Participants			How the effects of capsaicin on burning mouth syndrome were measured	Diagnostic method for burning mouth syndrome	Groups under investigation	Therapeutic strategy employed	Treatment period	Adverse and side effects	Statistical analysis	Results
Study design	Source of sample	Sample size	Mean age in years								
Petruzzi, M et al., (2004) Italy Triple‐blind clinical study	Not found	50 patients (14 men and 36 women)	20–40 years old	Visual analogue scale (VAS)	Patients with a burning sensation in the tongue or other oral tissue in the absence of local lesions	Group 1: Capsaicin capsules	Group 1: 0.25% tid capsules for 4 weeks	4 weeks of treatment	Minor adverse events were reported, characterised by gastric pain, with no other specific events occurring	Fisher's exact test *p* < 0.05	The values obtained in the VAS were lower in patients treated with capsaicin compared to the control group
						Group 2: Placebo, with capsules with the same shape/taste/smell/colour	Group 2: Capsules with same shape/taste/smell/colour for the same period of time				
Marino, R., (2010) Italy Prospective study	Department of Oral Pathology and Oral Medicine, University of Milan	56 patients (10 men and 46 women)	62 years old ±9.8	Visual analogue scale (VAS)	Patients who complained of a burning, stinging, or painful sensation in the mouth, in the absence of changes in the appearance of the oral mucosa or any local or systemic diseases	Group 1: Oral rinse with capsaicin	Group 1: Oral rinse with capsaicin 250 mg of pepper powder emulsified in 50 mL of water three times a day	Participants were followed for 8 weeks during the intervention (study I) and for a further 2 months after treatment (study II)	No adverse effects were observed in the patients, and the treatment was well tolerated	Wilcoxon test *p* < 0.05	A reduction in pain symptom scores on the VAS was observed in all patients who received capsaicin when compared to the control group. The efficacy of capsaicin was similar to that of alpha‐lipoic acid and lysozyme‐lactoperoxidase. Most patients treated with capsaicin also experienced improvement in burning sensation in the mouth, bitter taste in the mouth, tingling sensation and subjective thickening of saliva, with only 1 patient reporting worsening of symptoms
						Group 2: Alpha‐lipoic acid	Group 2: Alpha‐lipoic acid 400 mg twice a day				
						Group 3: Lysozymelactoperoxidase in the form of an oral rinse	Group 3: Lysozymelactoperoxidase in the form of an oral rinse five times a day				
						Group 4: Control, received boric acid in the form of an oral rinse	Group 4: Control, received boric acid 0.05 g in the form of an oral rinse dissolved in 100 mL of distilled water, three times				
Silvestre Donat et al., (2011)	Not found	23 patients (19 women and 4 men)	72.65 ± 12.10 years old	Visual analogue scale (VAS)	Patients with discomfort that was present daily for at least 6 months	Group A: 0.02% Capsaicin	Group A: Capsaicin 0.02% was administered three times daily with rinses performed for approximately 30 s in volumes of 15 mL in both groups	Participants received one week of treatment, followed by one week without intervention (washout), and then underwent another week of treatment in the opposite group	An intense burning sensation was reported by about one‐third of participants during the application of the capsaicin rinse and for up to 20 min after the procedure	Wilcoxon test *p* < 0.05	The group that used the mouthwash with capsaicin showed lower scores on the VAS scale, while no difference was observed in the placebo group
Valence						Group B: Placebo					
Prospective double‐blind											
Jankovskis et al., (2021) Latvia Controlled clinical trial	Institute of Stomatology (Stomatoloģijas institūts)	89 patients (8 men and 81 women)	89 years old	Visual analogue scale (VAS) and salivary flow	Persistent or intermittent oropharyngeal symptoms, with possible periods of remission and exacerbation throughout the day, without the presence of clinically or instrumentally detectable lesions	Group 1: Received vitamin B complex and zinc supplement	Zinc supplement—1 pill of 500 mg and vitamin B complex supplement—1 pill of 370 mg	3 weeks of treatment	It did not report whether or not there were any adverse or side effects	Wilcoxon test *p* < 0.05	The use of capsaicin was able to reduce pain levels on the VAS scale compared to the control group. Regarding salivary flow, unstimulated salivary flow increased from 0.14 to 0.225 mL/min after treatment. Stimulated salivary flow showed a slight reduction, from 0.94 to 0.91 mL/min, although some patients reported perceiving an improvement
					Furthermore, there are no identifiable local or systemic factors that explain the condition, such as oral diseases, medications, trauma, or physicochemical agents	Group 2: Received vitamin B complex and zinc supplement +0.02% capsaicin topical rinse	If zinc and vitamin B therapy failed to relieve symptoms, a 0.02% capsaicin rinse was used 3 times a day for 30 s after meals				

### 2.5 Data Extraction

The extracted data was organised into standardised tables and included the following information: authors and year of publication, country, study design, sample characteristics (size, gender, age range, location and study groups); method used for BMS diagnosis, groups evaluated, treatment time, form of capsaicin administration, frequency of capsaicin use, area affected by BMS, therapeutic strategy employed, statistical analysis, side effects and main results obtained. No additional statistical calculations were necessary. Due to the heterogeneity of the intervention protocols, the outcomes evaluated and the follow‐up periods, the results were synthesised descriptively. No subgroup analyses or meta‐regression were performed. Pain intensity measured by the Visual Analogue Scale was considered a continuous outcome; mean differences reported by the included studies were used to describe the magnitude and direction of treatment effects in the narrative synthesis.

The results of the included studies were organised and presented in standardised tables and figures containing information on methodological characteristics, interventions, outcomes assessed, adverse effects and main results. The study selection process was presented using a flowchart according to the PRISMA 2020 recommendations.

### 2.6 Risk of Bias

The risk of bias assessment of the studies was performed independently by two examiners (A.P.A.S. and Z.A.S.). In case of disagreement, a third reviewer (R.D.S.‐R.) was consulted to assist in the decision‐making process. The qualifier was chosen according to the design of the studies analysed. Version 2 of the Cochrane risk‐of‐bias tool for randomised trials (RoB 2) was used for randomised trials [20], and for non‐randomised studies, the Risk of Bias in Non‐randomised Studies of Interventions (ROBINS‐I) was used [21].

### 2.7 GRADE

For the analysis of evidence certainty, a narrative synthesis of the data collected from the clinical studies selected for the review was performed, observing the key aspect of pain scale reduction in randomised and non‐randomised studies. In this regard, the characteristics of the target population, follow‐up time and group criteria for risk of bias, inconsistency, indirectness and imprecision were evaluated, concluding with a grouped description of the results. The GRADE (Grading of Recommendations, Assessment, Development and Evaluation), a classification system for the level and strength of evidence for health recommendations, was used to calculate the expected absolute effect and summarise the evidence. The mean difference was used as an estimate of the effect for the Evidence Profile. When serious or extremely serious concerns regarding bias, inconsistency, indirectness, imprecision and publication bias are identified, the certainty of the evidence drops by one or two levels. The level of evidence tends to improve when the effect of all plausible confounding factors is minimised or when it suggests a spurious effect. Furthermore, publication bias could not be formally assessed because only four studies met the eligibility criteria, precluding reliable assessment of reporting bias.

## 3 Results

### 3.1 Search Results

**FIGURE 1:**
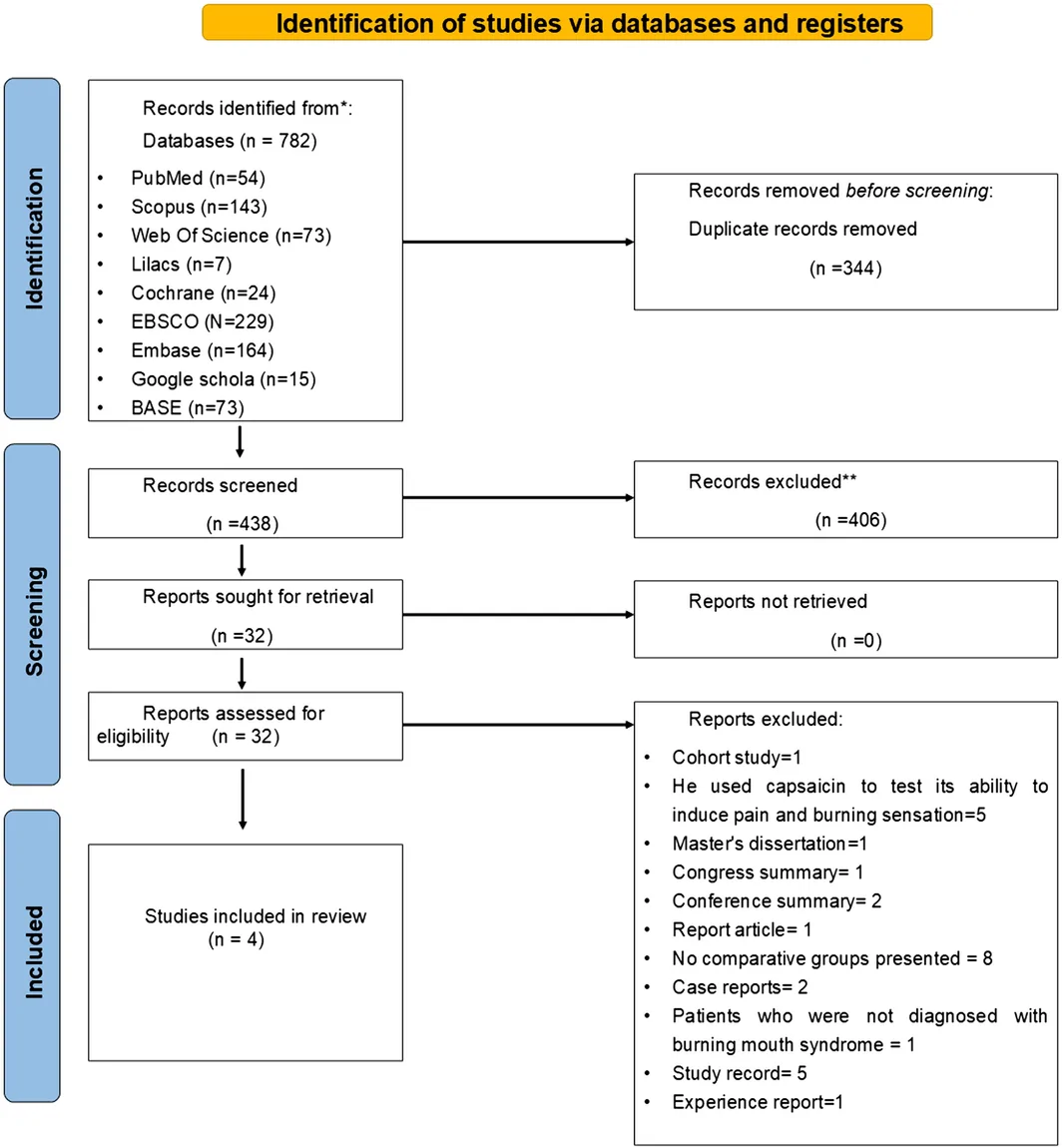
Flowchart of the study selection process according 2020.

A total of 782 articles were retrieved. Of these, 344 were excluded for being duplicates, 406 based on the title and abstract analysis, resulting in 32 articles selected for full reading. From the full reading, 1 article was excluded for being a cohort study, 5 for using capsaicin to test its ability to induce pain and burning sensation, 1 master's dissertation, 1 congress abstract, 2 conference abstracts and 1 report article, 8 articles that not include a group of comparison, 2 case reports, 1 article involving patients who were not diagnosed with BMS, 5 study registries, 1 experience report article, observed in Table S2. In the end, 4 articles were selected for review as shown in Figure 1.

### 3.2 Characteristics of the Studies

The types of studies identified were prospective double‐blind randomised controlled trials (*n* = 1) [16], triple‐blind randomised controlled trials (*n* = 1) [15], prospective randomised controlled trials (*n* = 1) [22] and non‐randomised controlled trials (*n* = 1) [23]. Of the 4 studies, 2 were from Italy [15, 22], 1 from Spain [16] and 1 from Latvia [23].

The total group of participants was 218 patients with a mean age of 63.41 years. Regarding gender, there were 182 women and 36 men. All participants were diagnosed with BMS.

Among the selected articles, the diagnostic method for BMS adopted in both the study by Marino et al., (2010) [22] and that of Petruzzi et al., (2004) [15] was the presence of oral burning in the tongue or other tissues of the mouth, in the absence of local lesions. In addition, Marino et al., (2010) [22] took into account the absence of other local or systemic diseases. Silvestre et al., (2012) [16] considered the report of persistent daily discomfort for at least 6 months. In turn, in the study by Jankovskis et al., (2021) [23], patients with persistent or intermittent oropharyngeal symptoms were included, who presented periods of remission and exacerbation during the day, without the presence of clinically or instrumentally detectable lesions, nor identification of local or systemic factors.

Regarding the study groups, 2 articles were clinical trials in which the control group was a placebo [15, 16], in 1 study the control was supplementation with vitamin B and zinc supplementation [23] and in 1 study the control was boric acid diluted in distilled water [22].

About the method by which capsaicin was administered, 3 articles investigated its application as a mouthwash [16, 22, 23] and 1 in capsule form [15]. The concentrations of capsaicin used in the mouthwash were 0.02% (*n* = 2) [16, 23] and 0.000354% (*n* = 1) [22], while the article that used the capsule form investigated the concentration of 0.25% [15]. In the studies by Silvestre et al., (2012) [16] and Jankovskis et al., (2021) [23], the mouthwash was applied for approximately 30 s, while in the study by Marino et al., (2010) [22] this application time was not reported. The frequency of use was 3 times a day (*n* = 4) [15, 16, 22, 23] with a treatment time of one week (*n* = 1) [16], 3 weeks (*n* = 1) [23], 4 weeks (*n* = 1) [15] and 8 weeks (*n* = 1) [22].

The area affected by burning mouth syndrome was specified in only one of the included studies [23], which identified the tip of the tongue, followed by the sides.

Two studies mentioned the immediate effects after using capsaicin, reporting that about a third of participants experienced an intense burning sensation for up to 20 min [16] and the presence of gastric pain [15]. One study reported that none of the participants experienced adverse effects [22], while 1 did not mention any adverse events associated with the capsaicin intervention [23].

The measurement of pain intensity reported by patients was the main outcome described in the 4 selected studies, and in all of them, this was assessed using the Visual Analogue Scale (VAS). In 3 studies [16, 22, 23], the results indicated that capsaicin administration promoted pain reduction on the first day of use. In these articles, it was described that before treatment, pain scores measured on the VAS ranged from 2 to 7, and that after capsaicin use, these scores ranged from 0 to 5. However, in 1 study [15], the results showed that capsaicin failed to relieve pain in all patients undergoing treatment.

Only 2 studies described results relating to other symptoms of BMS. Jankovskis et al., (2021) [23] demonstrated that rinsing with capsaicin provided improvement in unstimulated salivary flow in 7 of the 10 patients evaluated, with an increase in mean salivary values from 0.14 to 0.225 mL/min after treatment. In contrast, there was a slight reduction in mean levels of stimulated salivary flow, which went from 0.94 to 0.91 mL/min. Despite this, 7 patients still reported perceived improvement in relation to this parameter. In turn, the article by Marino et al., (2010) [22] carried out a more comprehensive evaluation, including, in addition to the burning and tingling sensation, the bitter taste in the mouth and subjective thickening of saliva. In this study, most patients treated with capsaicin (10 of 14 patients at the end of phase I of the study and 6 of 9 patients at the end of phase II of the study) showed improvement in these symptoms, with only one patient reporting worsening at the end of both studies.

### 3.3 Risk of Bias in Included Studies

**FIGURE 2:**
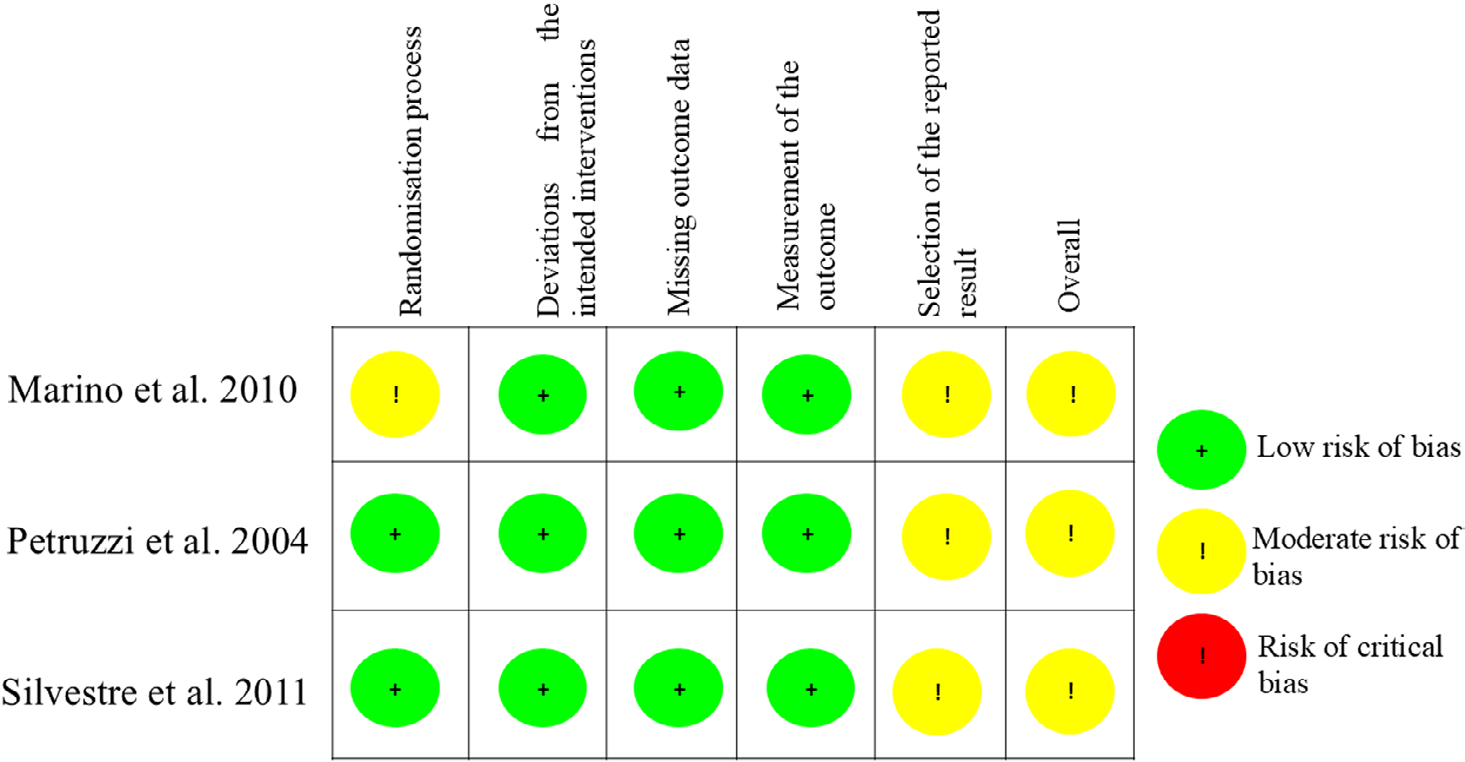
Risk of bias assessment in randomised clinical trials using the RoB 2 tool.

**FIGURE 3:**
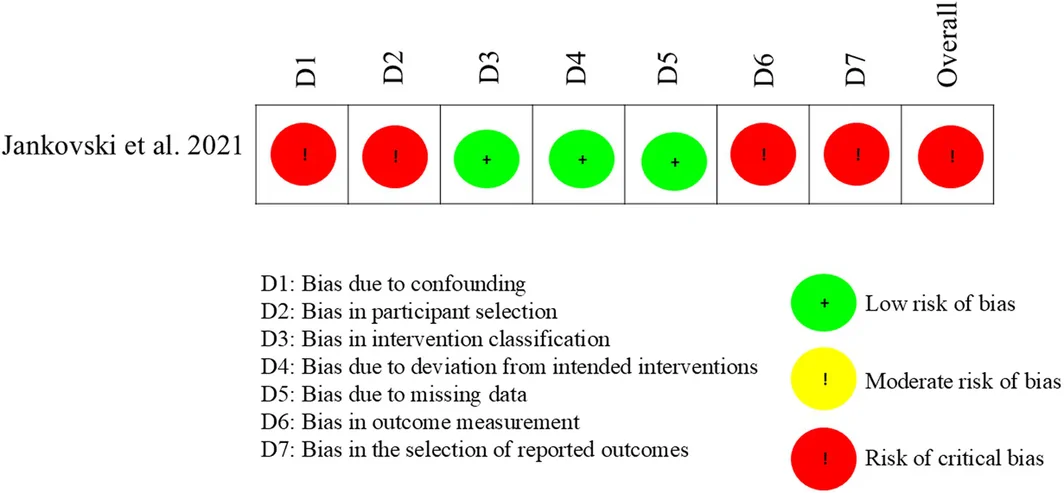
Risk of bias assessment in non‐randomised controlled clinical trials using the ROBINS‐I tool.

Three randomised clinical trials were assessed for risk of bias using the Cochrane RoB 2 tool [20]. Of these, all three were classified as having a moderate risk of bias (Figure 2). The single controlled clinical trial assessed using the ROBINS‐I [21] tool was considered to have a critical risk of bias (Figure 3).

### 3.4 Analysis of the Certainty of Evidence (GRADE)

Due to the small number of included studies and the substantial clinical and methodological heterogeneity among interventions, outcome assessments and follow‐up periods, no formal investigation of heterogeneity or sensitivity analyses were conducted. Therefore, the certainty of the evidence was assessed through a narrative GRADE approach.

The analysis of certainty of narrative evidence, using the GRADE Tool, showed a low level of certainty of evidence for randomised studies and a very low level of certainty of evidence for the non‐randomised study (Table 2). As for the randomised studies, the three studies identified presented problems of inconsistency in the direction of results between studies [15, 16, 22], as well as two studies [15, 16] also presented problems in the criterion of imprecision, given the absence of a confidence interval. As for the same criterion of changes in the pain scale, referring to the non‐randomised study [23], it presented serious problems related to confounding factors, participant selection and outcome measurement, as well as the criterion of indirectness, since the study's objective was not aligned with the main question of the review. The criterion of imprecision was also considered serious due to the absence of a confidence interval.

**TABLE 2:** Analysis of the certainty of evidence (GRADE) regarding changes in the pain scale through the treatment of burning mouth syndrome with capsaicin.

Capsaicin compared to others treatments/Placebo for burning mouth syndrome						
Certainty assessment						Summary of findings
Participants (studies) Follow‐up	Risk of bias	Inconsistency	Indirectness	Imprecision	Overall certainty of evidence	Impact
Changes in pain scale—Visual analogue scale in randomised studies (follow‐up: 120 days)						
165 (3 RCTs)	Not serious	Serious^a^	Not serious	Serious^b^	⨁⨁◯◯ Low^a^, ^b^	The results of the three studies show that the use of capsaicin can reduce the degree of pain on a statistically significant scale. However, these results vary in terms of magnitude of effect and the methodology used
Changes in pain scale—Visual analogue scale in Non‐randomised study (follow‐up: 60 days)						
50 (1 non‐randomised study)	Very serious^c^	Not serious	Very serious^d^	Serious^e^	⨁◯◯◯ Very low^c^, ^d^, ^e^	The study shows an association between prior supplementation with zinc and vitamin B, followed by treatment with capsaicin, resulting in pain reduction. However, there is no group treated with capsaicin alone

^a^ Studies point to the same outcome, but show great variability in the magnitude of the effect.

^b^ Only the study by Marino et al., 2010 provides data for measuring the confidence interval; the other studies (Silvestre et al., 2012; Petruzzi et al., 2004) present statistical and sample results but without sufficient data for measuring the confidence interval.

^c^ The study presented serious concerns regarding the analysis of risk of bias within the parameters of confounding factors, participant selection and outcome measurement.

^d^ The study prioritises the evaluation of systemic supplementation with other compounds.

^e^ The study does not disclose the confidence interval; neither does it provide the means and standard deviation values that would allow the confidence interval to be measured.

## 4 Discussion

This systematic review evaluated the available evidence on the efficacy of capsaicin in the management of BMS. Overall, the studies suggest that capsaicin may reduce pain scores associated with BMS. However, the interpretation of these findings should be cautious due to the limited methodological quality of the available studies. Risk of bias assessment using ROBINS‐I and RoB‐2 tools indicated a moderate risk of bias in randomised clinical trials and a critical risk in the non‐randomised study. Consistently, the GRADE certainty of evidence analysis indicated a low level of certainty for randomised studies and a very low level for the non‐randomised study.

BMS is a chronic condition characterised by the absence of evident causative lesions or local and systemic conditions that could explain the symptoms primarily affecting middle‐aged or postmenopausal women [1, 24]. The pathophysiology of this syndrome has not yet been fully elucidated, but it is considered multifactorial and involves peripheral and central neuropathic alterations, hormonal dysfunctions and sensory alterations of the oral mucosa [25, 26, 27, 28]. Histopathological evidence demonstrates atrophy of nerve fibres in the tongue and alterations in the innervation of the taste buds, suggesting the involvement of small fibre neuropathy [26, 27]. In this context, interventions capable of modulating peripheral nociceptive transmission, such as capsaicin, have been investigated as potential therapeutic strategies.

Capsaicin acts as an agonist of transient receptor potential vanilloid 1 (TRPV1), an ion channel expressed in sensory nerve fibres and involved in the perception of thermal and nociceptive stimuli [29]. Repeated or prolonged activation of these receptors promotes neuronal desensitisation, resulting in reduced excitability of nociceptive fibres and decreased pain transmission [30, 31]. TRPV1 receptor is present in the fungiform papillae of the tongue, structures that may show increased density in patients with BMS [25]. Thus, the modulation of these receptors by capsaicin could represent a relevant mechanism for reducing the painful symptoms observed in these patients.

In the 4 selected studies, approximately 83% of the 218 patients evaluated were women. This finding is consistent with the epidemiology of BMS, which has a higher prevalence in women, especially after menopause [24]. Reduced levels of ovarian steroids may contribute to alterations in peripheral neural function and pain modulation, reducing the neuroprotective effects of these hormones on the nervous system [28]. Experimental models in ovariectomised animals also demonstrate structural alterations in the lingual mucosa associated with hormonal deficiency, including epithelial thinning [32, 33].

Furthermore, in these studies, capsaicin was administered 3 times a day, with treatment durations ranging from 1 to 8 weeks. The need for repeated applications can be explained by the transient nature of the TRPV1 receptor desensitisation process. Although the initial activation of these receptors may induce an acute nociceptive response, discomfort and short‐lived erythema, after repeated or prolonged applications of capsaicin, there is a reduction or even absence of neuronal response to subsequent nociceptive stimuli, the so‐called functional desensitisation and even to capsaicin itself, termed pharmacological desensitisation [30, 34]. This mechanism can be used to explain a relevant issue highlighted in the selected studies, which is the initial discomfort frequently reported after capsaicin application, followed by progressive improvement of symptoms with continued use. The intensity of this burning sensation varies among individuals and, in some cases, compromises adherence to treatment [35]. Silvestre et al., (2012) [16] reported that, of a total of 23 patients investigated, one third presented a transient burning sensation for about 20 min after using the mouthwash, and two participants dropped out due to the initial discomfort caused. A possible strategy to improve treatment tolerability involves the use of topical anaesthetics before applying capsaicin, as reported by Yosipovitch et al., (1999) [36], who demonstrated that pretreatment with EMLA (2.5% lidocaine and 2.5% prilocaine) significantly reduced the induced burning sensation. Although this approach has not been investigated in any of the clinical trials included in our review, it may represent a viable strategy to improve treatment adherence and suggests the need for investigation in future studies.

Topical administration with mouthwash was the most frequently used approach among the included studies [16, 22, 23], while systemic administration in capsules was used in only one study [15]. In general, studies using mouthwashes report an average reduction of approximately 2 points in VAS and, although previous evidence suggests similar efficacy between topical and systemic routes, topical application may present important clinical advantages, including faster onset of action and lower incidence of systemic adverse effects [15, 35, 36, 37]. This may explain the predominance of studies investigating topical administration, especially considering that capsaicin administered orally is absorbed in the gastrointestinal tract [38] and metabolised in the liver [39]. Petruzzi et al., (2004) [15] reported the occurrence of gastric irritation in 8 of 50 patients treated with capsaicin capsules. An experimental study in mice demonstrated that systemic administration of capsaicin can induce dose‐dependent gastrointestinal changes, including villous rupture, intestinal mucosal desquamation, infiltration of inflammatory cells and loss of mucus‐producing goblet cells in the colon [40]. These changes appear to be associated with the induction of an inflammatory response mediated by increased pro‐inflammatory cytokines such as IL‐1β, TNF‐α and IL‐10 [32, 41, 42].

Despite this, the efficacy of topical administration has been shown to be limited in some studies. Silvestre et al., (2012) [16] reported that capsaicin mouthwash induced a transient burning sensation and did not promote complete remission of symptoms in any patient, in addition to not demonstrating a sustained effect over time. Jankovskis et al., (2021) [23] observed a reduction in pain and burning sensation after using capsaicin, but patients continued to experience a progressive increase in symptoms throughout the day. Marino et al., (2010) [22], although they did not observe adverse effects among patients, and the treatment was well tolerated, repeated applications of capsaicin were necessary due to its short duration of action.

When considered together, these findings suggest that topical administration may present a more favourable safety profile when compared to the systemic route, particularly because it does not involve the gastrointestinal effects associated with oral absorption. However, this possible benefit seems to occur at the expense of a limited duration of the analgesic effect and the need for frequent reapplications of capsaicin. Thus, although current data indicate that topical formulations may represent a clinically safer alternative for the treatment of BMS, especially in patients more susceptible to systemic adverse effects, it is not yet possible to establish definitive clinical recommendations due to the small sample size, methodological heterogeneity and short follow‐up period of the available studies.

The capsaicin concentrations evaluated in the included studies ranged from 0.000354% to 0.25%, all associated with a reduction in pain scores. Systemic administration of 0.25% capsaicin for four weeks, as described by Petruzzi et al., (2004) [15], resulted in significant improvement in patients with high initial pain scores (VAS between 8 and 10). On the other hand, patients with moderate initial scores (VAS between 4 and 7) did not show significant improvement. Regarding topical administration, Silvestre et al., (2012) [16] observed an average reduction of two points on the VAS scale after one week of treatment with a mouthwash containing 0.02% capsaicin. Similar results were reported by Jankovskis et al., (2021) [23], who observed an average reduction of 1.5–2 points after three weeks of treatment. Marino et al., (2010) [22], compared capsaicin with alpha‐lipoic acid and lysozyme‐lactoperoxidase, demonstrating symptomatic improvement in all therapeutic groups, with no significant differences between them.

In addition to burning and pain symptoms, BMS may be associated with symptoms such as dysgeusia, xerostomia, altered sensitivity of the oral mucosa and psychological impairment [9, 14]. In the study by Jankovskis et al., (2021) [23], capsaicin promoted an increase in unstimulated salivary flow and improved stimulated salivary flow. This effect can be explained by the activation of the TRPV1 receptor, expressed not only in sensory neurons, but also in non‐neuronal cells, including salivary glands [43, 44]. Capsaicin favours salivary secretion by increasing the paracellular permeability of the tight junction [45]. Furthermore, Marino et al., (2010) [22] reported improvement in symptoms associated with BMS, such as tingling, bitter taste and a sensation of thick saliva after the use of capsaicin. These results are similar to those observed by Jørgensen and Pedersen, (2017) [46], who demonstrated improvement in symptoms with the application of capsaicin gel at concentrations of 0.01% or 0.025%, with no statistically significant difference between the gels.

The trigeminal pathway is responsible for transmitting afferent information from the oral cavity [47]. It is known that the main target receptor of capsaicin, TRPV1, is expressed in the trigeminal ganglia and plays a fundamental role in physiological sensory perception and response to environmental stimuli [47]. In this context, the improvement in the tingling sensation and the perception of thick saliva may be related to the modulation of this receptor, since repeated stimulation by capsaicin can lead to desensitisation of the afferent fibres responsible for the transmission of nociceptive and sensory signals involved in the symptomatology of burning mouth syndrome [48]. As for the action of capsaicin on bitter taste, it may be related not only to the desensitisation of sensitive nociceptors present in the trigeminal nerve, but also to the modulation of taste. Green et al., (2003) [49] demonstrated that pretreatment with capsaicin significantly reduced the perception of quinine bitterness in the anterior portion of the tongue, however these effects varied between different taste regions, since in the region of the circumvallate papillae, there was no significant desensitisation of bitterness for any of the substances evaluated, suggesting that this difference may be related to the greater expression of the TRPV1 receptor in the fungiform papillae of the tongue [25].

Quality assessment demonstrated that randomised clinical trials were evaluated as having a moderate risk of bias, while the non‐randomised clinical trial presented a critical risk of bias. The GRADE assessment results showed that the selected studies ranged from low to very low levels of certainty. The main risk of bias in the randomised studies included in this systematic review was related to the method of measuring the effects of capsaicin, since most of the included studies used exclusively the VAS, restricting the analysis to only one measurement method, while in the non‐randomised study it was due to the way in which patients to be subjected to capsaicin were allocated. Regarding the certainty of evidence analysis, this was compromised by the variability of the capsaicin doses used in each of the included studies, which makes it difficult to compare the results, in addition to few studies reporting or providing means for evaluating the confidence interval.

The methodological quality of the included studies limits the strength of the conclusions. The main source of bias in the randomised clinical trials was related to the measurement of outcomes, since most used exclusively the VAS scale for pain assessment. Even though widely used in clinical studies, this scale is based on the patient's subjective perception and can be influenced by psychological and individual factors. The heterogeneity in doses, administration methods and treatment duration also made direct comparison between studies difficult. Although the results of this study should be interpreted with caution, we believe that the data presented can serve for the development of new clinical studies in order to validate the reported findings.

## 5 Conclusion

Despite the limited number of available studies and the low certainty of the identified evidence, the results of this systematic review suggest that capsaicin may contribute to the reduction of painful symptoms in patients with burning mouth syndrome (BMS). However, due to the low certainty of the available evidence, clinical recommendations cannot yet be established. The heterogeneity of therapeutic protocols and the methodological limitations of the included studies restrict the robustness of these conclusions. Therefore, well‐designed randomised clinical trials with larger samples, standardisation of doses and administration protocols and the use of multiple clinical assessment instruments are needed to provide more consistent evidence on the efficacy and safety of capsaicin in the management of BMS.

## Author Contributions

**Ana Paula de Andrade Silva:** investigation, writing – original draft, methodology, writing – review and editing, formal analysis, conceptualization. **Ian Wesley Rocha dos Santos:** investigation, methodology, formal analysis, writing – original draft. **Wallacy Watson Pereira Melo:** writing – original draft, methodology, investigation, formal analysis. **Deborah Ribeiro Frazão:** writing – original draft, writing – review and editing, methodology, formal analysis. **Rafael Rodrigues Lima:** writing – review and editing, funding acquisition, methodology, formal analysis. **Renata Duarte de Souza‐Rodrigues:** conceptualization, writing – original draft, methodology, validation, writing – review and editing, formal analysis, project administration, supervision. **José Mário Matos‐Sousa:** writing – original draft, writing – review and editing, methodology, formal analysis. **Zuleni Alexandre da Silva:** investigation, writing – original draft, methodology, formal analysis.

## Funding

This work was supported by CAPES—Coordenação de Aperfeiçoamento de Pessoal de Nível Superior, FAPESPA—Fundação Amazônia de Amparo a Estudos e Pesquisas, Conselho Nacional de Desenvolvimento Científico e Tecnológico (CNPq) and received grant number 312275/2021‐8. Also, this research was funded by PROCAD Amazônia—CAPES (23038.005350/2018‐78).

## Conflicts of Interest

The authors declare no conflicts of interest.

## Supporting information


**Data S1:** Prisma checklist.
**Table S1:** Search key.
**Table S2:** Articles excluded after reading in full and reasons for exclusions.

## Data Availability

The data that supports the findings of this study are available in the Supporting Information of this article.
