# Supplementary material for: Is Capsaicin Effective in Relieving the Symptoms of Burning Mouth Syndrome? A Systematic Review
Source: J Oral Rehabil. 2026 Jul 24;53(10):1985–96. doi: 10.1111/joor.70275 (PMC13583394; doi:10.1111/joor.70275)
Supplement: Supplementary file 1 [file JOOR-53-1985-s001.docx]

**Is capsaicin effective in relieving the symptoms of burning mouth syndrome? A systematic review**

**Short running title:** Capsaicin and relief of Burning Mouth Syndrome

Ana Paula de Andrade Silva^1^, Ian Wesley Rocha dos Santos^1^, Zuleni Alexandre da Silva^1^, Deborah Ribeiro Frazão^1^, José Mário Matos-Sousa^1^, Wallacy Watson Pereira Melo^1^, Rafael Rodrigues Lima^1^, Renata Duarte de Souza-Rodrigues^1*^

1 Laboratory of Functional and Structural Biology, Institute of Biological Sciences, Federal University of Pará, Belém 66075-110, Brazil; [ana.andrade.silva@ics.ufpa.br](mailto:ana.andrade.silva@ics.ufpa.br)

1 Laboratory of Functional and Structural Biology, Institute of Biological Sciences, Federal University of Pará, Belém 66075-110, Brazil; [zulenilisboa@gmail.com](mailto:zulenilisboa@gmail.com)

1 Laboratory of Functional and Structural Biology, Institute of Biological Sciences, Federal University of Pará, Belém 66075-110, Brazil; [deborahfrazao@hotmail.com](mailto:deborahfrazao@hotmail.com)

1 Laboratory of Functional and Structural Biology, Institute of Biological Sciences, Federal University of Pará, Belém 66075-110, Brazil; [ian.santos@ics.ufpa.br](mailto:ian.santos@ics.ufpa.br)

1 Laboratory of Functional and Structural Biology, Institute of Biological Sciences, Federal University of Pará, Belém 66075-110, Brazil; [jms.ufpa@outlook.com](mailto:jms.ufpa@outlook.com)

1 Laboratory of Functional and Structural Biology, Institute of Biological Sciences, Federal University of Pará, Belém 66075-110, Brazil; [wallacy.melo@ics.ufpa.br](mailto:wallacy.melo@ics.ufpa.br)

1 Laboratory of Functional and Structural Biology, Institute of Biological Sciences, Federal University of Pará, Belém, Brazil; [rafalima@ufpa.br](mailto:rafalima@ufpa.br)

1 Laboratory of Functional and Structural Biology, Institute of Biological Sciences, Federal University of Pará, Belém, Brazil; [renataduarte@ufpa.br](mailto:renataduarte@ufpa.br).

*Corresponding author: Renata Duarte de Souza-Rodrigues, Augusto Corrêa Street, n. 01, Guamá. 66075-110. Belém, Pará, Brazil, Laboratory of Functional and Structural Biology, Institute of Biological Sciences Federal University of Pará, +55 91 98020-5559, renataduarte@ufpa.br

**Acknowledgments**

The authors would like to thank the Federal University of Pará for technical and scientific support and this work was supported by CAPES - Coordenação de Aperfeiçoamento de Pessoal de Nível Superior, FAPESPA - Fundação Amazônia de Amparo a Estudos e Pesquisas, Conselho Nacional de Desenvolvimento Científico e Tecnológico (CNPq) and received grant number 312275/2021-8. Also, this research was funded by PROCAD Amazônia - CAPES (23038.005350/2018-78).

**Supplementary** Prisma checklist

| **Section and Topic** | **Item #** | **Checklist item** | **Location where item is reported** |
| --- | --- | --- | --- |
| **TITLE** | | |  |
| Title | 1 | Identify the report as a systematic review. | Page 1, lines 1 to 2 |
| **ABSTRACT** | | |  |
| Abstract | 2 | See the PRISMA 2020 for Abstracts checklist. | Page 2, lines 40 to 59 |
| **INTRODUCTION** | | |  |
| Rationale | 3 | Describe the rationale for the review in the context of existing knowledge. | Pages 2 – 3-4, lines 63 to 113 |
| Objectives | 4 | Provide an explicit statement of the objective(s) or question(s) the review addresses. | Page 3- 4 lines 113 to 119 |
| **METHODS** | | |  |
| Eligibility criteria | 5 | Specify the inclusion and exclusion criteria for the review and how studies were grouped for the syntheses. | Page 5, lines 142 to 155 |
| Information sources | 6 | Specify all databases, registers, websites, organisations, reference lists and other sources searched or consulted to identify studies. Specify the date when each source was last searched or consulted. | Pages 4, lines 129 to 132 |
| Search strategy | 7 | Present the full search strategies for all databases, registers and websites, including any filters and limits used. | Pages 4, lines 134 to 137 |
| Selection process | 8 | Specify the methods used to decide whether a study met the inclusion criteria of the review, including how many reviewers screened each record and each report retrieved, whether they worked independently, and if applicable, details of automation tools used in the process. | Page 5 lines 142 to 167 |
| Data collection process | 9 | Specify the methods used to collect data from reports, including how many reviewers collected data from each report, whether they worked independently, any processes for obtaining or confirming data from study investigators, and if applicable, details of automation tools used in the process. | Page 5, lines 157 to 167 |
| Data items | 10a | List and define all outcomes for which data were sought. Specify whether all results that were compatible with each outcome domain in each study were sought (e.g. for all measures, time points, analyses), and if not, the methods used to decide which results to collect. | Page 5-6, lines 169 to 185 |
|  | 10b | List and define all other variables for which data were sought (e.g. participant and intervention characteristics, funding sources). Describe any assumptions made about any missing or unclear information. | Page 5-6, lines 170 to 175 |
| Study risk of bias assessment | 11 | Specify the methods used to assess risk of bias in the included studies, including details of the tool(s) used, how many reviewers assessed each study and whether they worked independently, and if applicable, details of automation tools used in the process. | Page 6, lines 186 to 193 |
| Effect measures | 12 | Specify for each outcome the effect measure(s) (e.g. risk ratio, mean difference) used in the synthesis or presentation of results. | Page 6, lines 175 to 180 |
| Synthesis methods | 13a | Describe the processes used to decide which studies were eligible for each synthesis (e.g. tabulating the study intervention characteristics and comparing against the planned groups for each synthesis (item #5)). | Page 5, lines 143 to 155 |
|  | 13b | Describe any methods required to prepare the data for presentation or synthesis, such as handling of missing summary statistics, or data conversions. | Page 6, lines 175 to 180 |
|  | 13c | Describe any methods used to tabulate or visually display results of individual studies and syntheses. | Page 6, lines 181 to 185 |
|  | 13d | Describe any methods used to synthesize results and provide a rationale for the choice(s). If meta-analysis was performed, describe the model(s), method(s) to identify the presence and extent of statistical heterogeneity, and software package(s) used. | Page 6, lines 175 to 185 |
|  | 13e | Describe any methods used to explore possible causes of heterogeneity among study results (e.g. subgroup analysis, meta-regression). | Page 6, lines 177 to 180 |
|  | 13f | Describe any sensitivity analyses conducted to assess robustness of the synthesized results. | --- |
| Reporting bias assessment | 14 | Describe any methods used to assess risk of bias due to missing results in a synthesis (arising from reporting biases). | Page 6, lines 187 to 193 |
| Certainty assessment | 15 | Describe any methods used to assess certainty (or confidence) in the body of evidence for an outcome. | Page 6-7, lines 195 to 209 |
| **RESULTS** | | |  |
| Study selection | 16a | Describe the results of the search and selection process, from the number of records identified in the search to the number of studies included in the review, ideally using a flow diagram. | Page 7, lines 212 to 219 |
|  | 16b | Cite studies that might appear to meet the inclusion criteria, but which were excluded, and explain why they were excluded. | Page 7, lines 218 to 219 |
| Study characteristics | 17 | Cite each included study and present its characteristics. | Pages 7-9, lines 221 to 278 |
| Risk of bias in studies | 18 | Present assessments of risk of bias for each included study. | Page 9, lines 281 to 284 |
| Results of individual studies | 19 | For all outcomes, present, for each study: (a) summary statistics for each group (where appropriate) and (b) an effect estimate and its precision (e.g. confidence/credible interval), ideally using structured tables or plots. | ---- |
| Results of syntheses | 20a | For each synthesis, briefly summarise the characteristics and risk of bias among contributing studies. | Page 9, lines 281 to 284 |
|  | 20b | Present results of all statistical syntheses conducted. If meta-analysis was done, present for each the summary estimate and its precision (e.g. confidence/credible interval) and measures of statistical heterogeneity. If comparing groups, describe the direction of the effect. | --- |
|  | 20c | Present results of all investigations of possible causes of heterogeneity among study results. | Page 6, lines 175 to 176 |
|  | 20d | Present results of all sensitivity analyses conducted to assess the robustness of the synthesized results. | Page 9, lines 280 to 301 |
| Reporting biases | 21 | Present assessments of risk of bias due to missing results (arising from reporting biases) for each synthesis assessed. | Page 9, lines 280 and 284 |
| Certainty of evidence | 22 | Present assessments of certainty (or confidence) in the body of evidence for each outcome assessed. | Page 9, lines 285 to 301 |
| **DISCUSSION** | | |  |
| Discussion | 23a | Provide a general interpretation of the results in the context of other evidence. | Pages 9-10, lines 303 to 310 |
|  | 23b | Discuss any limitations of the evidence included in the review. | Page 14, lines 445 to 453 |
|  | 23c | Discuss any limitations of the review processes used. | Page 14, lines 445 to 453 |
|  | 23d | Discuss implications of the results for practice, policy, and future research. | Pages 14, lines 456 to 465 |
| **OTHER INFORMATION** | | |  |
| Registration and protocol | 24a | Provide registration information for the review, including register name and registration number, or state that the review was not registered. | Page 4, lines 123 to 125 |
|  | 24b | Indicate where the review protocol can be accessed, or state that a protocol was not prepared. | Page 4, line 125 to 126 |
|  | 24c | Describe and explain any amendments to information provided at registration or in the protocol. | --- |
| Support | 25 | Describe sources of financial or non-financial support for the review, and the role of the funders or sponsors in the review. | Page 1, lines 28 to 33 |
| Competing interests | 26 | Declare any competing interests of review authors. | Page 15, lines 473 to 475 |
| Availability of data, code and other materials | 27 | Report which of the following are publicly available and where they can be found: template data collection forms; data extracted from included studies; data used for all analyses; analytic code; any other materials used in the review. | Page 15, lines 473 to 475 |

*From:* Page MJ, McKenzie JE, Bossuyt PM, Boutron I, Hoffmann TC, Mulrow CD, et al. The PRISMA 2020 statement: an updated guideline for reporting systematic reviews. BMJ 2021;372:n71. doi: 10.1136/bmj.n71. This work is licensed under CC BY 4.0. To view a copy of this license, visit <https://creativecommons.org/licenses/by/4.0/>

**Supplementary Table 1.** Search key

| **I** | - Capsaicin - 8 methyl n vanillyl 6 nonenamide - abc-pflaster - adlea - alacapsin - algrx 4975 - algrx4975 - axsain - biozone - capsaicine - capser - capsicaine - capsicin - capsidol - capsig - capsina - captrix - capzasin-hp - casacine - cgs 200 - cgs200 - cntx 4975 - cntx 4975 05 - cntx 497505 - cntx4975 - cntx497505 - decenoic acid vannillylamide - dolenon - dolorac - moment (capsaicin) - n (4 hydroxy 3 methoxybenzyl) 8 methylnon 6 enamide - n (4 hydroxy 3 methoxybenzyl) 8 methylnon trans 6 enamide - n [ (4 hydroxy 3 methoxyphenyl) methyl] 8 methylnon 6 enamide - ngx 1998 - ngx 4010 - ngx1998 - ngx4010 - picasum - qutenza - sensedol - sensocaps - styptysat - trans 8 methyl n vanillyl 6 nonenamide - trans capsaicin - transacin - trixaicin - vanillylamide of 9 methylnon 6 enoic acid - zacin - zostrix - zostrix cream - zostrix-hp |
| --- | --- |
| O | - Burning Mouth Syndrome - burning mouth - burning mouth pain - burning mouth syndrome - burning oral pain - burning pain in the mouth - burning pain in the oral cavity - mouth burning sensation - oral burning - oral burning pain - oral burning sensation - oral dysaesthesia - oral dysesthesia - oral pyrosis - orolingual paraesthesia - oropyrosis - stomatopyrosis - syndrome, burning mouth - Glossalgia - Glossalgias - Glossodynia - Glossodynias - Glossopyrosis - Glossopyroses - Stomatodya |

**Supplementary Table 2:** Articles excluded after reading in full and reasons for exclusions

| **Reference** | **Reason for exclusion** |
| --- | --- |
| 1. Silvestre-Rangil, J., Silvestre, F. J., Tamarit-Santafé, C., & Bautista, D. (2011). Burning mouth syndrome: correlation of treatment to clinical variables of the disease. Medicina oral, patologia oral y cirugia bucal, 16(7), e890–e894. https://doi.org/10.4317/medoral.17224 | Cohort study |
| 2. Yilmaz, Z., Renton, T., Yiangou, Y., Zakrzewska, J., Chessell, I. P., Bountra, C., & Anand, P. (2007). Burning mouth syndrome as a trigeminal small fibre neuropathy: Increased heat and capsaicin receptor TRPV1 in nerve fibres correlates with pain score. *Journal of clinical neuroscience : official journal of the Neurosurgical Society of Australasia*, *14*(9), 864–871. https://doi.org/10.1016/j.jocn.2006.09.002 | Evaluated capsaicin for pain induction |
| 3. Yilmaz, Z., Egbuniwe, O., & Renton, T. (2016). The Detection of Small-Fiber Neuropathies in Burning Mouth Syndrome and Iatrogenic Lingual Nerve Injuries: Use of Quantitative Sensory Testing. *Journal of oral & facial pain and headache*, *30*(2), 87–98. https://doi.org/10.11607/ofph.1531 | Evaluated capsaicin for pain induction |
| 4. Naka, K., Orabović, I., Mrla, A., Vrbanović, E., Alajbeg, I., Boucher, Y., & Alajbeg, I. (2022). Obrazac odgovora na bol promijenjen je u bolesnika sa sindromom pekućih usta. *Acta stomatologica Croatica*, 203-203. | Evaluated capsaicin for pain induction |
| 5. Gøkhan, M. A., Sørensen, E. S., & Baad-Hansen, L. (2023). Role of dairy proteins in the reduction of capsaicin-induced oral burning pain. *Physiology & behavior*, *259*, 114036. https://doi.org/10.1016/j.physbeh.2022.114036 | Evaluated capsaicin for pain induction |
| 6. Just, T., Steiner, S., & Pau, H. W. (2010). Oral pain perception and taste in burning mouth syndrome. Journal of oral pathology & medicine : official publication of the International Association of Oral Pathologists and the American Academy of Oral Pathology, 39(1), 22–27. https://doi.org/10.1111/j.1600-0714.2009.00824.x | Evaluated capsaicin for pain induction |
| 7. FRÉO, Bianca. *Clinical study activity of capsaicin in patients with Burning Mouth Syndrome*. 2008. Dissertação (Mestrado em Odontologia) — Faculdade de Odontologia, Universidade de São Paulo, São Paulo, 2008. | Master's thesis |
| 8. Lauritano D, Papagna R, Caccaniga G, Baldoni M, Bascones A. *New therapeutical approaches with capsaicin: a double blind study in a group of patients with burning mouth syndrome.* 2003. | Congress summary |
| 9. Fréo B, Sugaya NN, Pellegrini VD. *Topical capsaicin treatment of burning mouth syndrome: a pilot study*. In: Proceedings of the General Session of the International Association for Dental Research; 2012. | Conference summary |
| 10. Rivetti E, Colangelo G, Brini AT, Groppetti A, Spadari F. *Sindrome della bocca bruciante con capsaicina topica*. Doctor OS. 2005;16(2):75 | Conference summary |
| 11. Azzi, L., Costantino, D., Tettamanti, L., Tagliabue, A., & Spadari, F. (2013). Management of burning mouth syndrome: a clinical experience. Annali di stomatologia, 4(Suppl 2), 6–7. | Report article |
| 12. Jørgensen, M. R., & Pedersen, A. M. (2017). Analgesic effect of topical oral capsaicin gel in burning mouth syndrome. *Acta odontologica Scandinavica*, *75*(2), 130–136. https://doi.org/10.1080/00016357.2016.1269191 | Does not present a comparison group |
| 13. Jørgensen , M R & Pedersen , A M L 2010 , ' Effect of Oral Capsaicin Gel on Burning Mouth Syndrome ' , Oral Diseases , vol. 16 , pp. 549 . | Does not present a comparison group |
| 14. Spadari, F., Azzi, L., Porrini, M., Rossi, M., Tagliabue, A., & Tettamanti, L. (2014). *Burning mouth syndrome and topical capsaicin: A clinical experience*. In *Oral Diseases* (Vol. 20, Suppl. 2, pp. 40.13–40.13). | Does not present a comparison group |
| 15. Spanemberg, J. C., Rodriguez-Archilla, A., Ceballos-Salobreña, A., Coppola, M. C., & de Araújo, L. M. A. (2011). Burning mouth syndrome: topical application of 0.002% capsaicin (capsicum frutescens l). *Health Care Magazine*, *9*(28). | Does not present a comparison group |
| 16. Ricken, C. M., Péder, S. N. S., Kamikawa, D. S., Pieralisi, N., Chicarelli, M., & Tolentino, E. S. (2021). *Evaluation of a protocol for topical application of capsaicine gel 0.025% in the management of burning mouth syndrome correlating its impact on quality of life*. International Journal of Odontostomatology, 15(2), 443–448. https://doi.org/10.4067/S0718-381X2021000200443 | Does not present a comparison group |
| 17. Azzi, L., Croveri, F., Pasina, L., Porrini, M., Vinci, R., Manfredini, M., Tettamanti, L., Tagliabue, A., Silvestre-Rangil, J., & Spadari, F. (2017). A burning therapy for burning mouth syndrome: preliminary results with the administration of topical capsaicin. *Journal of biological regulators and homeostatic agents*, *31*(2 Suppl 1), 89–95. | Does not present a comparison group |
| 18. Espinosa, S. & Lopez Jornet, Pia & Ros, R.. (2004). Burning mouth syndrome: Clinical study about efficacy of topical capsaicin application. Pilot study. Avances en odontoestomatologia. 20. 297-304. | Does not present a comparison group |
| 19. ZBOROWSKI, J.; KAROLEWICZ, B.; DOŁOWACKA-JÓŹWIAK, A.; et al. Topical and mucoadhesive administration of capsaicin in the burning mouth syndrome treatment. *Journal of Clinical Medicine*, 2026;15(2):780. doi:10.3390/jcm15020780. | Does not present a comparison group |
| 20. Castro, A., Sampaio, C., & Pereira, A. (2014). BURNING MOUTH SYNDROME: HOMEMADE SOLUTION OF PEPPER-AN ALTERNATIVE?: WIP-0395. *Pain Practice*, *14*, 77. | Case report |
| 21. IMAI, U.; BETSUBE, S. A case in which combined therapy with Rikkosan and capsaicin was effective for pain suspected to be caused by oral burning syndrome. *Itami to Kampo (Pain and Kampo)*, 2024. | Case report |
| 22. Scardina, GA, Carini, F., Valenza, V., & Messina, P. (2006). Topical capsaicin application and axon reflex vasodilatation of the tongue: Neurogenic involvement in burning mouth syndrome. *Methods and Findings in Experimental and Clinical Pharmacology, 28(10), 707-712.* | Involving patients who were not diagnosed with BMS |
| 23. The Effect of Capsaicin on Burning Moth. (Irct20220117053748N), 2022. | study registries |
| 24. Neurogenic Mechanisms in Burning Mouth Syndrome. (NCT00875537), 2009. | study registries |
| 25. Burning Mouth Syndrome - New Diagnostic Criteria and Treatment. (NCT05059418), 2021. | study registries |
| 26. Effect of Treatments on Pain and Quality of Life in Individual With Burning Mouth Syndrome. (NCT06040190), 2023. | study registries |
| 27. The efficacy of topical capsaicin in reducing symptoms in patients with burning mouth syndrome*.* Registry of Clinical Trials (ACTRN12608000235303), 2008. | study registries |
| 28. Spice R, Hagen NA. Capsaicin in burning mouth syndrome: titration strategies. *J Otolaryngol*. 2004;33(1):53–54. | Experience report article |
